# Plasmapheresis Is Associated With Better Renal Outcomes in Lupus Nephritis Patients With Thrombotic Microangiopathy: A Case Series Study

**DOI:** 10.1097/MD.0000000000003595

**Published:** 2016-05-06

**Authors:** Qiu-Yu Li, Feng Yu, Fu-De Zhou, Ming-Hui Zhao

**Affiliations:** From the Department of Medicine, Renal Division, Peking University First Hospital; Institute of Nephrology, Peking University; Key laboratory of Renal Disease, Ministry of Health of China; Key Laboratory of CKD Prevention and Treatment, Ministry of Education of China (Q-YL, FY, F-DZ, M-HZ); Department of Nephrology, Peking University International Hospital (FY); and Peking-Tsinghua Center for Life Sciences, Beijing, PR China (M-HZ).

## Abstract

The aim of this study was to evaluate the efficacy of plasmapheresis in patients with lupus nephritis-combined thrombotic microangiopathy (TMA) in a Chinese cohort.

Clinical and therapeutic data of patients with lupus nephritis–combined TMA were collected retrospectively. A comparison between those with and without plasmapheresis was performed.

Seventy patients with renal biopsy-proven TMA in lupus nephritis were treated with conventional combined corticosteroid and immunosuppressive agents as induction therapy, 9 of the 70 patients received additional plasmapheresis. The plasmapheresis group presented with more severe SLE and renal activity indices, including a significant higher ratio of neurologic disorder (*P* = 0.025), lower level of platelet count (*P* = 0.009), higher value of serum creatinine (*P* = 0.038), higher percentage of anti-cardiolipin antibodies positive (*P* = 0.001), and higher Systemic Lupus Erythematosus Disease Activity Index scores (*P* = 0.012), than that of the nonplasmapheresis group. However, the plasmapheresis group had a significant higher rate of remission and a lower ratio of treatment failure than that of the nonplasmapheresis group (*P* = 0.03). As the baseline data were significantly different between the 2 groups, the propensity score match was further designed to avoid retrospective bias. After re-analysis, the plasmapheresis group still had a significant higher rate of remission and a lower ratio of treatment failure than that of the nonplasmapheresis group (*P* = 0.018). More importantly, the plasmapheresis group had significant less composite endpoints than that of the nonplasmapheresis group (*P* = 0.005).

Our study suggested that additional plasmapheresis on conventional induction therapy may benefit patients with lupus nephritis-combined TMA, which warrants further explorations.

## Abstract 2

Supplemental Digital Content is available in the text

## INTRODUCTION

Thrombotic microangiopathy (TMA) is characterized by microangiopathic hemolytic anemia, thrombocytopenia, acute kidney injury, and/or fever, and/or neurologic impairment.^1^ It was consisted of several diseases including thrombotic thrombocytopenic purpura (TTP), hemolytic uremic syndrome (HUS), malignant hypertension, and some autoimmune disorders like systemic lupus erythematosus (SLE), etc.^2–4^

Lupus nephritis-combined TMA was not rare in previous studies,^5–14^ and it was up to 17% in our recent study.^15^ The high mortality and poor renal outcomes were noted in patients with lupus nephritis-combined TMA.^6^ There were no standardized guidelines for the treatment of patients with lupus nephritis-combined TMA, although 2012 KDIGO Clinical Practice Guideline for Glomerulonephritis suggested that “patients with systemic lupus and thrombotic thrombocytopenic purpura (TTP) receive plasmapheresis as for patients with TTP without systemic lupus. (2D),” which was indicated as “research recommendations.”^16^ However, the published studies lacked detailed descriptions to demonstrate the efficacy of plasmapheresis in the patients.^5,6,17–19^

Herein, this retrospective study evaluated the efficacy of plasmapheresis in patients with lupus nephritis-combined TMA in our lupus nephritis cohort, and we further reviewed and analyzed published reports in the literature.

## METHODS

### Patients Selection

**FIGURE 1:**
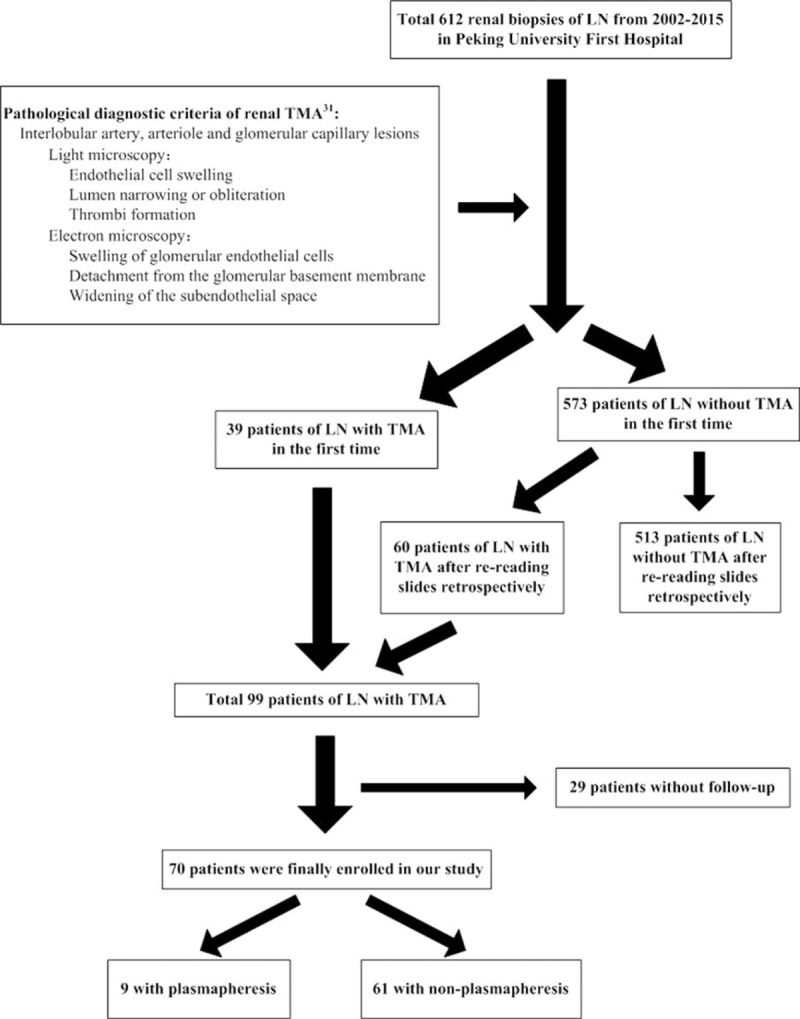
Inclusion criteria of lupus nephritis combined TMA patients and the design of the study. LN = lupus nephritis, TMA = thrombotic microangiopathy.

We retrospectively reviewed the medical records of 612 patients with renal biopsy-proven lupus nephritis, who were admitted to Peking University First Hospital between January 2002 and May 2015. The inclusion criteria and study flow chart of patients with lupus nephritis-combined TMA were described in Figure 1. The diagnosis of SLE was established according to the criteria of the American Rheumatism Association.^20^ TMA was characterized by microangiopathic hemolytic anemia, thrombocytopenia, acute renal injury, and/or fever and/or neurologic impairment. Renal TMA was confirmed by biopsied pathological findings as described later.

Informed consent was obtained from each patient. The research was in compliance with the Declaration of Helsinki. The design of this work was approved by the local ethical committees of Peking University First Hospital (No. 2012[470]).

### Clinical Evaluation

The disease activity was assessed by the Systemic Lupus Erythematosus Disease Activity Index (SLEDAI).^21,22^ The following clinical items were collected and analyzed: sex, fever, malar rash, photosensitivity, oral ulcer, alopecia, arthritis, serositis, neurologic disorder, anemia, leukocytopenia, thrombocytopenia, hematuria, and leukocyturia.

Medical insurance status and annual family income of the patients were also investigated.

### Baseline Immunosuppressive Therapy for Lupus Nephritis Patients Based on KDIGO Guideline

The therapy recommended for Class III and Class IV lupus nephritis includes initial and maintenance courses.^23^ The standard regimens of initial therapy include cyclophosphamide or mycophenolate mofetil in combination with glucocorticoids. All the subjects with remission were converted to a less-toxic regimen (mycophenolate mofetil or azathioprine) combined with low-dose glucocorticoid regimens for maintence of remission.

Patients with Class II or V lupus nephritis could receive glucocorticoids and immunosuppressants as dictated by the persistent nephrotic proteinuria or extrarenal manifestations of SLE.

### Plasmapheresis Procedures

#### Routine Plasmapheresis

With dual vascular access being applied as described,^24^ heparin or low-molecular-weight heparin was used as anticoagulant in plasma exchange procedures. In theory, 1.0 to 1.5 plasma volumes (PVs), which was the target volume of the procedure, may lead to about 20% to 40% of the residual relative concentration^25^. And an OP08 filter (Asahi Kasei Corporation, Tokyo, Japan) was used as the first filter. In most cases, each patient was removed 1 plasma volume, and 5% albumin and fresh frozen plasma (FFP) were applied to conduct 100% replacement. Almost all the patients, who underwent plasmapheresis, were dispensed 1 ampule (10 mL) of 5% calcium gluconate, promethazine, (5 mL, 12.5 mg), and hexadecadrol (5–10 mg) to prevent hypocalcemia^26–28^. After each procedure, the parameters were displayed on the screen of the instrument, including whole blood flow rate, procedure time, plasma volume procedured, plasma volume removed, volume of applied heparin, or low-molecular-weight heparin, were recorded. With an average plasma flow rate of up to 30 mL/min, the flow of blood was set to 120 mL/min. During plasmapheresis, waste plasma was discarded intermittently. Before and after the procedure, the blood of each patient was instantly sampled as well.

#### Double Filtration Plasmapheresis (DFPP)

This process was as similar as a previous report.^29^ In briefly, 1.0 or 1.5 volumes of plasma were processed in each session of double filtration plasmapheresis (DFPP). The OP08 filter (Asahi Kasei Corporation) was used for plasma separation, and the EC30W filter (Asahi Kasei Corporation) was used for plasma fractionation. Native blood was pumped into the OP08 filter, and then the filtered plasma was pumped into the EC30W filter. In the latter process, the albumin was separated from the larger plasma molecules. The blood volume flow rate was set as 120 mL/min and the mean plasma flow rate could reach up to 30 mL/min. When the pressure on the EC30W filter reached the threshold value, we used 1000 mL normal saline to flush the filter.

#### Laboratory Assessment

We collected the following patients’ items before treatment for further analysis as our previous report^30^: complete blood count, plasma lactate dehydrogenase, liver enzymes, peripheral blood smear, urine analysis, serum creatinine, serum antinuclear antibodies (ANA), anti-double-stranded DNA (ds-DNA) antibodies, anti-extractable nuclear antigen (ENA) antibodies, anti-cardiolipin antibodies and C3.

#### Renal Histopathology

The renal biopsy specimens were examined by light microscopy, direct immunofluorescence, and electron microscopy techniques in accordance with our previous reports.^31^ All the samples were reviewed by 2 experienced pathologists (double-blind method) based on the recommendation of the International Society of Nephrology and Renal Pathology Society (ISN/RPS) 2003 lupus nephritis classification system.^32^ The pathologists classified and scored the biopsies separately, especially for the activity indices (AI), chronicity indices (CI), and renal TMA evaluations.^15,33–35^ Differences in scoring between the pathologists were resolved by re-reviewing the biopsies and thus reaching a consensus.

### Definitions of Treatment Response for TMA and Endpoints for the Patients

The response to therapy includes complete remission, partial remission, and treatment failure, which was as same as previous works by Geerdink et al.^36^ Relapse was defined as an episode of acute TMA >30 days after remission.^37^ Our patients were followed up in outpatient clinic specified for lupus nephritis. The composite endpoints were defined as death, end-stage renal disease (ESRD), doubling of serum creatinine, or treatment failure.

### Literature Search

The therapeutic regimen and treatment responses for lupus nephritis combined with TMA patients were compared with different reports identified through systematic literature review, in accordance with previous report.^38^ Electronic searches were performed by using Medline, EMBASE, and Cochrane databases (1950 through July 2015) using relevant text words and medical subject headings that included all spellings of “thrombotic thrombocytopenic purpura,” “hemolytic uremic syndrome,” “thrombotic microangiopathy,” “SLE,” “lupus,” “lupus nephritis,” “treatment,” and “outcome.” The language of literature was limited to English.

### Statistical Analysis

Continuous variables were described as mean ± standard deviation (SD) or median (interquartile range [IQR]) and differences between groups were analyzed by using 2-factor analysis of variance test or nonparametric test. Categorized variables were described as percentage and analyzed by using the *χ*^2^ test. Hardy–Weinberg equilibrium was estimated using the *χ*^2^ goodness of fit test. Univariate and multivariate logistic regression analyses were used to assess survival. Results were expressed as odds ratio (OR) with 95% confidence intervals (CIs). Kaplan–Meier curves were used to analyze patients’ prognosis. The statistical analysis was performed with SPSS for Windows (version 12.0, SPSS Inc, Chicago, IL). A 2-tailed *P* value <0.05 was considered statistically significant.

SPSS software package (version 12.0, SPSS Inc) was employed for statistical analysis as in previous report.^38^ Quantitative parameters between groups were tested with *t* test (for normally distributed data) and results were presented as mean ± SD. Continuous variables were tested with nonparametric test (for data that were not normally distributed) and the results were described as median (IQR). Categorized variables were described as percentage and analyzed by using the *χ*^2^ test. Kaplan-Meier curves were used to analyze patients’ prognosis. Univariate and multivariate logistic regression analysis was used to evaluate renal survival. Results were expressed as OR with 95% CIs. A 2-tailed *P* value <0.05 was considered statistically significant. We also used propensity score adjustment to balance potential confounders with STATA.^39,40^

Logistic regression models were used to calculate the propensity score. Variables in the model included age (numerical value), sex (male vs female), economic status, baseline indices including serum creatinine value, anemia or not, acute renal failure or not, anticardiolipin antibody-positive or -negative, SLEDAI scores, and treatment regimen except plasmapheresis.
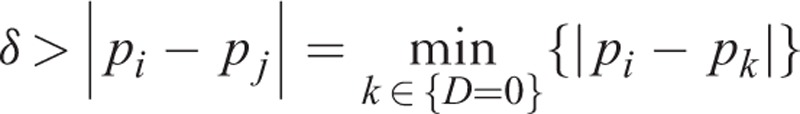


We matched each case to 1 control on the basis of the propensity score according to Barbara Siamese's recommendation (University College London and Institute for Fical Study) (http://www.doc88.com/p-7098910122782.html). For a prespecified δ ≤0.0001, treated unit i is matched to that nontreated unit j according to the formula. If none of the nontreated units is within δ from treated unit i, i is left unmatched.

## RESULTS

### General Data of Patients With Lupus Nephritis Combining with TMA

Among the 70 patients enrolled in the study, 17 were male and 53 were female, with an average age of 29.71 ± 10.23 years at presentation (Table 1). The causes of TMA in the lupus nephritis patients were as follows: 2 patients with TTP-HUS, 5 with anti-phospholipid antibody syndrome (APS), 8 with malignant hypertension, 3 with scleroderma, and the other 52 presented with isolated renal TMA changes.

**TABLE 1:**
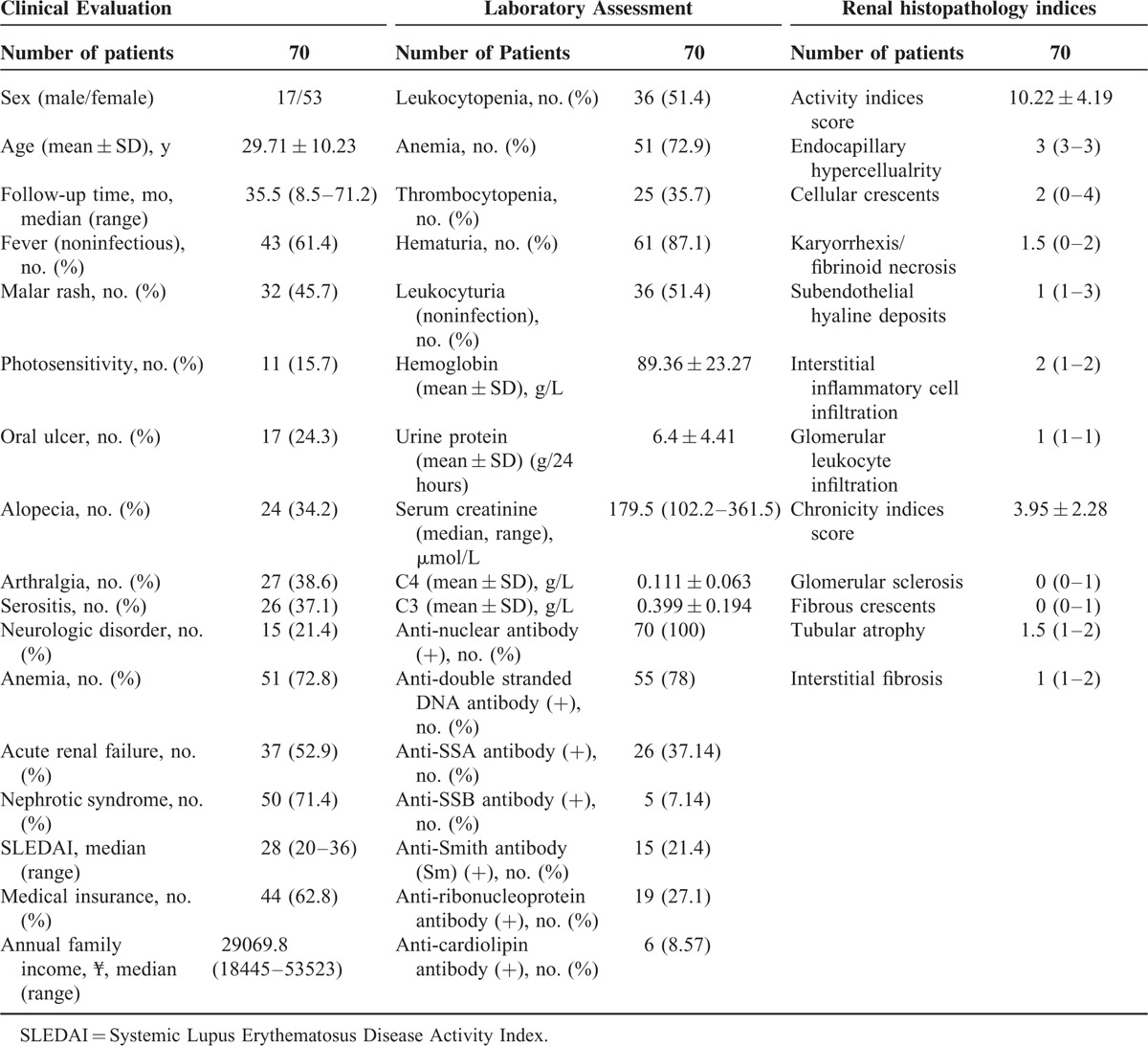
General Data of Patients With Lupus Nephritis Combining With TMA

According to the 2003 classification of lupus nephritis, 2 patients were classified as Class II, 6 patients as Class III (including 4 with Class III + V), 53 as Class IV (1 as Class IV-segmental [IV-S] and 52 as Class IV-global [IV-G], including 8 with Class IV + V), and 7 as Class V.

The treatment algorithm was listed as following: all of the patients received oral prednisone therapy. The majority of patients completed treatment with monthly intravenous cyclophosphamide (48/70) (600–800 mg/month). The other patients received mycophenolate mofetil (8/70) or leflunomide (5/70). 68.6% (48/70) of patients received methylprednisolone pulse. Nine patients received plasmapheresis, including 2 with TTP-HUS, 4 with APS, 1 with malignant hypertension, and 2 with isolated renal TMA. The detailed descriptions of the 9 patients with plasmapheresis treatment were shown in the Table 2.

**TABLE 2:**
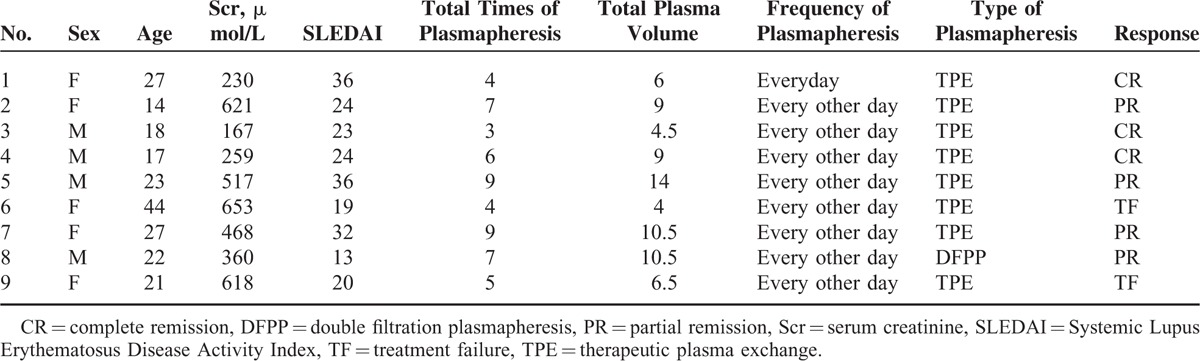
Clinical Data of 9 Lupus Nephritis Patients Receiving Plasmapheresis Treatment

The patients were followed up for nearly 3 years. In total, 20 patients got clinical remission, including complete remission and partial remission, and 50 patients presented with treatment failure. Fifty patients reached composite endpoints, including that 1 was dead, 34 entered ESRD, and 15 reached doubling of serum creatinine.

We further compared the clinical characteristics of patients with plasmapheresis treatment or not.

### Comparison of Clinical Data and Outcome Between Patients With and Without Plasmapheresis Treatment (Unmatched Groups)

The clinical features of the patients in the 2 groups were listed in Table 3. There were no significant differences of the demographic data between the 2 groups. However, the group with plasmapheresis treatment presented with more severe SLE and renal disease active indices, including higher ratio of neurologic disorder (*P* = 0.025), lower level of platelet count (*P* = 0.009), higher value of serum creatinine (*P* = 0.038), higher percentage of positive serum anti-cardiolipin antibodies (*P* = 0.001), and higher SLEDAI scores (*P* = 0.012), than that of those in nonplasmapheresis group.

**TABLE 3:**
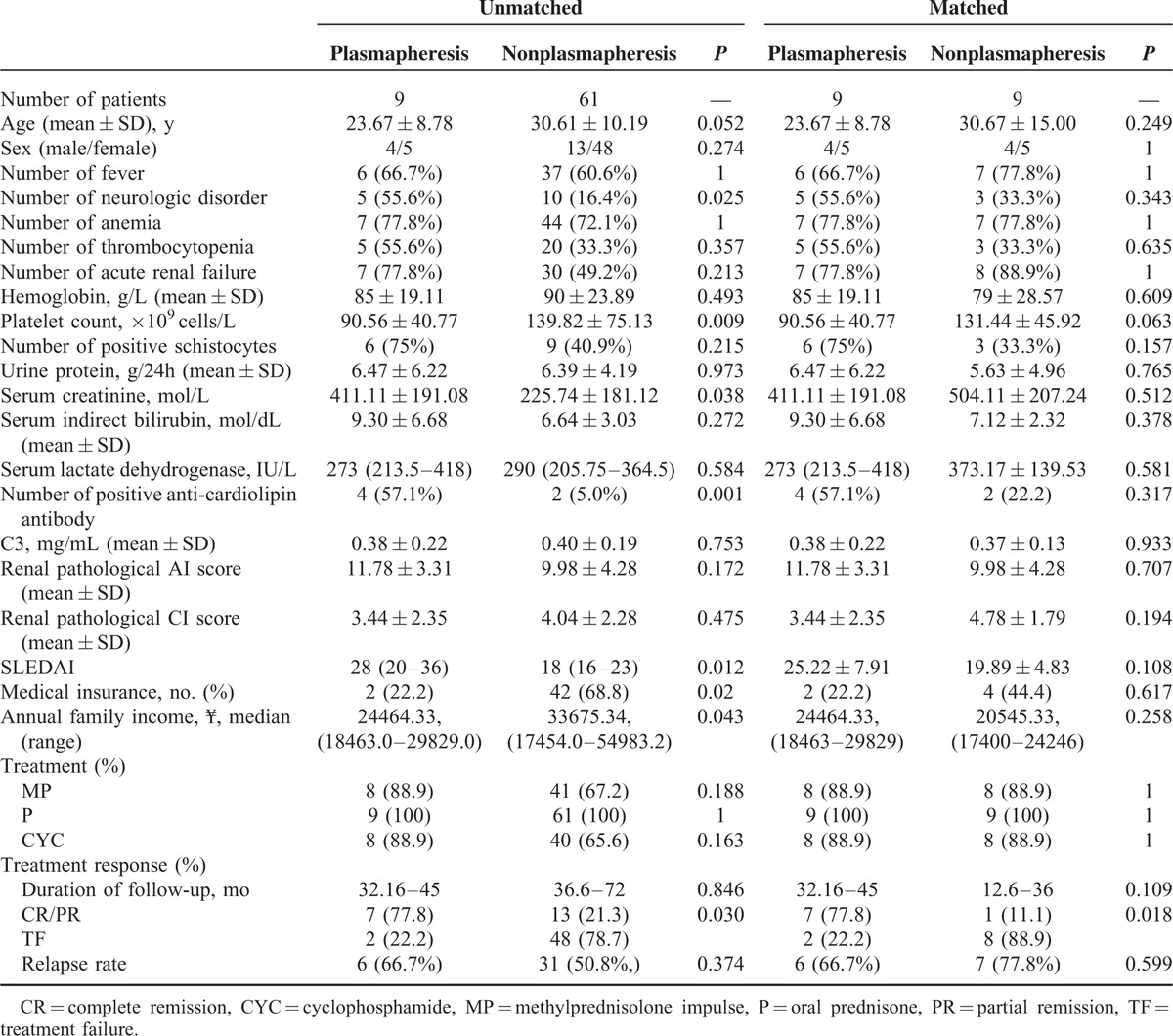
The Comparisons of Clinical Data Between Patients With and Without Plasmapheresis Treatment

As to economic status, we found that: the difference of the medical insurance ratio was significant between the 2 groups (2/9, 22.2% vs 42/61, 68.9%, *P* = 0.02); the difference of the average annual family income between the 2 groups was also significant (¥24464.33 vs ¥33675.34, *P* *=* 0.043).

There was no significant difference in the baseline treatment algorithm between the 2 groups. However, the group with plasmapheresis treatment presented with higher rate of remission and lower ratio of treatment failure compared with nonplasmapheresis group (*P* = 0.03).

**FIGURE 2:**
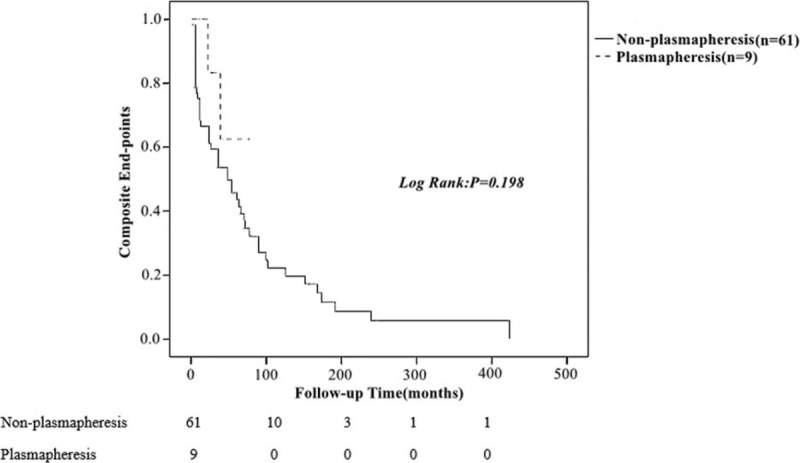
Comparison of the composite endpoints between unmatched patients with and without plasmapheresis treatment.

Regarding long-term composite endpoints during a similar follow-up time (average for nearly 3 years), there was no significant difference between the 2 groups (*P* = 0.198, Figure 2), in which 2 patients entered ESRD in the plasmapheresis group, of which 1 was dead, and 32 entered ESRD and 15 reached doubling of serum creatinine in the nonplasmapheresis group.

Using the log-rank test for univariate survival analysis of all the patients, we found that plasmapheresis was a beneficial factor (hazard ratio [HR]: 12.923, 95% CI: 2.392–69.807, *P* = 0.003), and anemia was a risk factor (HR: 0.22, 95% CI: 0.071–0.683, *P* = 0.009) for long-term outcome (Details in supplemental Table 1). When the candidate parameters (age, sex, serum creatinine, anemia, plasmapheresis, SLEDAI, and anticardilolipin antibody) entered into the multivariate analysis (details in supplemental Table 2), plasmapheresis was further proved to be independently beneficial factor associated with long-term outcomes (HR: 8.914, 95% CI: 3.028–26.247, *P* < 0.001).

A nested case–control analysis should be used to avoid retrospective bias, as our baseline data presented with significant difference between the 2 groups with or without plasmapheresis. Propensity score adjustment was then conducted as previous report^41^ as followings: propensity scores were calculated based on the predicted probabilities of the intitial treatment, and we then stratified cox models across the 5ths of the propensity score. It was further assumed that all related differences between the 2 groups with or without plasmapheresis were captured by the observables indices, including age, sex, economic status, baseline renal injury indices, SLEDAI scores, and treatment regimen except plasmapheresis. We selected from the non-plasmapheresis pool as control group in which the distribution of observed variables was as similar as possible to the distribution in the plasmapheresis group.

### Comparison of Clinical Data and Outcome between Patients With and Without Plasmapheresis Treatment (Matched Groups)

Table 3 also showed that the new 2 groups, including 9 patients with plasmapheresis treatment and 9 without plasmapheresis, were matched by age, sex, economic status, baseline indices, including anemia, acute renal failure, serum creatinine value, anticardiolipin antibody, SLEDAI scores, and treatment regimen except plasmapheresis.

The group with plasmapheresis treatment still presented with higher rate of remission and lower ratio of treatment failure compared with that of non-plasmapheresis group (*P* = 0.018), and the difference was more significant than that in unmatched groups.

**FIGURE 3:**
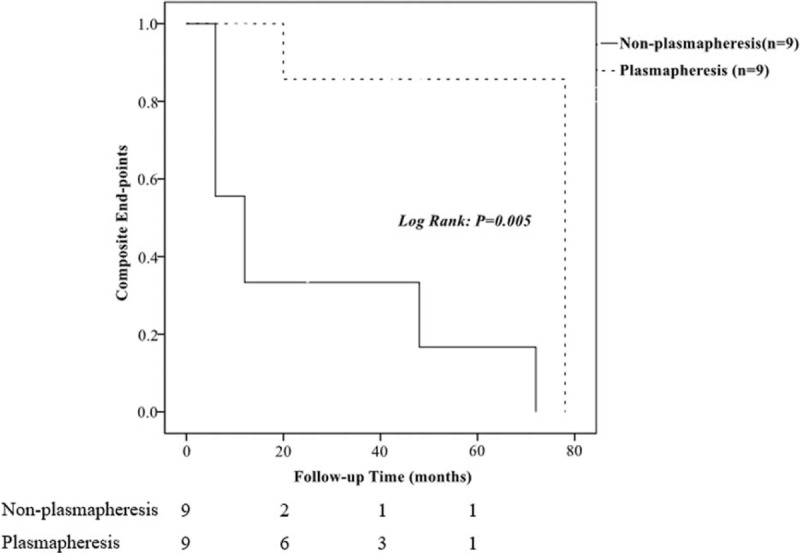
Comparison of the composite endpoints between matched patients with and without plasmapheresis treatment.

Regarding long-term outcome, there was significant difference between the 2 groups (*P* = 0.005, Figure 3), in which 2 patients entered ESRD in the plasmapheresis group, 1 was dead, and 7 patients entered ESRD in the nonplasmapheresis group.

## DISCUSSION

Our study found that the patients of lupus nephritis-combined TMA suffered high SLEDAI scores, including severe renal, hematologic, and neurologic disorders. As this is a retrospective study and most of the patients were with isolated renal TMA, only a few patients received plasmapheresis treatment (9 with plasmapheresis vs 61 with no plasmapheresis). The primary analysis showed that the group with plasmapheresis presented with a higher remission rate compared with that of the nonplasmapheresis group, even the former had more severe SLE and renal disease active indices. However, as the baseline of the 2 groups was unequivalent, it might bring the potential research bias and affect final analysis for endpoints. This could be indeed an inevitable problem with retrospective studies like ours, in which exposure and outcome might already occur at the time of individuals selected for study. To balance the baseline of the 2 groups, we matched our patients according to propensity for treatment estimated by a multivariate model removing potential confounding factors and we revealed a more significant difference in therapeutic response between patients with and without plasmapheresis. More importantly, the composite endpoints were fewer in patients with plasmapheresis after adjustment. Finally, the multivariate analysis confirmed that plasmapheresis was an independently beneficial factor associated with long-term outcomes in patients with lupus nephritis-combined TMA in our single-center experience. Unfortunately, there was a dearth of literatures in the area of describing the remission and renal survival rates using plasmapheresis in patients with lupus nephritis-combined TMA. Those publications lacked detailed information on frequency and total volume of plasmapheresis, let alone the type of plasmapheresis and relevant mechanism analysis^5,6,17–19^ (Table 4).

**TABLE 4:**
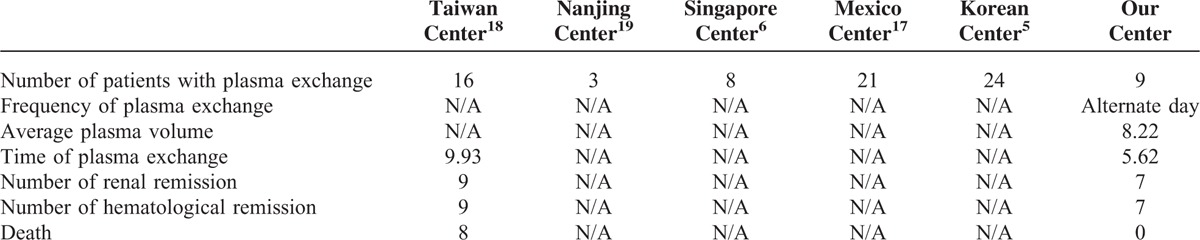
Comparisons Between the Patients in Present Study and Those From Previous Reports

The rationale for plasmapheresis in patients with lupus nephritis-combined TMA might be 3-fold: it removes a variety of offending plasma pathogens, such as autoantibodies, abnormal immunoglobulins, circulating immune complexes, abnormal coagulation factors, and circulating protein-bound toxic agents, it replaces deficient or defective biofunctional proteins, and it enables the administration of higher volumes of plasma.^42,43^ In technology, plasmapheresis is performed by using automated devices designed with specialized instruments for blood withdrawal, anticoagulation, separation, and blood return, as well as compartments for replacement fluid and separated substances. In our study, 8 patients experienced routine plasma exchange and 1 patient DFFP, both effectively reported in the literature.^44,45^ None of our patients experienced severe adverse events during plasmapheresis. Although DFFP had several advantages over routine plasmapheresis, such as it selectively removed macromolecules, no deficiency syndrome was observed, it did not require a replacement solution, less expensive, >1 plasma volume might be processed without increased cost or deficiency syndrome, it was a closed dead-end system with less chance of contamination and infection, and less volume shift was noticed, etc, the prospective clinical trials were needed. We suggested that plasmapheresis for treatment of lupus nephritis-combined TMA should be performed continually, such as once a day for 7 days, until the return of clinical remission of TMA manifestations, which was similar with previous study,^18^ and based on the experiences from TTP treatment.

As it was consisted of a group of diseases, including APS, TTP-HUS, scleroderma, malignant hypertension, drugs, pregnancy-induced syndrome, and abnormal complement activation-associated status, etc, the pathogenesis of lupus nephritis-combined TMA was complex and remained unclear. Given the heterogeneity of lupus nephritis-combined TMA, identifying mechanistic pathways common in most cases has diagnostic and therapeutical values.^46^ In the early literatures, most cases supported that serum anti-phospholipid antibodies might play an important role in the development of TMA in lupus, as lupus anticoagulants with or without anti-cardiolipin antibodies could be detected in the majority of patients with renal TMA-associated SLE.^46,47^ Moreover, lupus anticoagulants^48^ and IgG-anti-phospholipid antibodies^47^ were significantly associated with intraglomerular microthrombi formation in lupus nephritis. In a recent study conducted by Espinosa et al,^48^ 70% of the patients with thrombotic microangiopathic hemolytic anemia and positive serum antiphospholipid antibodies improved when receiving both plasmapheresis and immunosuppressants, compared with 34% of those without plasmapheresis treatment. Similarly, in our cohort, 4 patients with anti-phospholipid antibodies secondary to lupus received plasmapheresis and 3 achieved remission, which further supported the above theory.

However, among the total 70 patients in our study, only 5 patients were associated with anti-phospholipid antibody, and up to 52 (74.3%) presented with isolated renal TMA changes, which suggested other pathogenic factors for the development of TMA in lupus nephritis. Interestingly, recent studies,^31,47,49,50^ including ours, showed that there were evidences of complement overactivation both in circulation and kidneys loci in more than half of the lupus nephritis patients combined with TMA, which was irrespective of anti-phospholipid antibodies positive. Thus, it provided reasonable theory for most patients with lupus nephritis-combined TMA receiving plasmapheresis treatment, and also highlighted the potential use of anti-complement bioagents like Eculizumab, the anti-C5 antibody, which was proved to be efficacious in several recent case reports in the field.^51,52^

The advantage of our study is that it is the first detailed descriptive case series report on the use of plasmapheresis for lupus nephritis patients combined with TMA based on a well-defined cohort. However, there are some limitations: First, it was a retrospective study from a single center. Second, the case number of patients receiving plasmapheresis treatment was too small to compare effects between different settings of plasmapheresis, like albumin replacement, FFP replacement, or double filtration. Third, the mechanistic work was lacking in this article.

In conclusion, our retrospective study suggested that the treatment regimen of plasmapheresis might be effective in improving the recovery and renal outcomes of patients with lupus nephritis combined TMA. With the limitations of this retrospective analysis, the prospective multicenter explorations with larger sample size were needed.

## Supplementary Material

Supplemental Digital Content
