# Supplementary material for: Plasmapheresis Is Associated With Better Renal Outcomes in Lupus Nephritis Patients With Thrombotic Microangiopathy: A Case Series Study
Source: Medicine (Baltimore). 2016 May 6;95(18):e3595. doi: 10.1097/MD.0000000000003595 (PMC4863807; doi:10.1097/MD.0000000000003595)
Supplement: Supplemental Digital Content [file medi-95-e3595-s001.doc]

Supplemental Table 1. Univariate survival analysis of the patients

|  | **HR** | **95% CI** | | ***P* Value** |
| --- | --- | --- | --- | --- |
|  |  | **Lower** | **Upper** |  |
| Gender | 0.658 | 0.205 | 2.115 | 0.482 |
| Age | 0.538 | 0.184 | 1.575 | 0.258 |
| Fever | 0.625 | 0.214 | 1.822 | 0.389 |
| Neurologic disorder | 1.952 | 0.589 | 6.469 | 0.274 |
| Anemia | 0.22 | 0.071 | 0.683 | 0.009 |
| Thrombocytopenia | 0.1 | 0.604 | 10.344 | 0.206 |
| Proteinuria | 1.994 | 0.681 | 5.837 | 0.208 |
| Acute renal failure | 0.643 | 0.227 | 1.824 | 0.406 |
| SLEDAI | 2.426 | 0.809 | 7.279 | 0.114 |
| Serum creatinine | 0.783 | 0.273 | 2.244 | 0.648 |
| C3 | 2.105 | 0.733 | 6.046 | 0.167 |
| C4 | 1.6 | 0.49 | 5.222 | 0.436 |
| Anticardilolipin antibody | 4.125 | 0.698 | 24.386 | 0.118 |
| Anti-dsDNA antibody | 0.75 | 0.22 | 2.557 | 0.646 |
| Anti-Sm antibody | 0.886 | 0.246 | 3.2 | 0.854 |
| Activity indices (AI) score | 1.111 | 0.378 | 3.261 | 0.849 |
| Chronicity indices (CI) score | 1.163 | 0.388 | 3.492 | 0.787 |
| Plasmapheresis | 12.923 | 2.392 | 69.807 | 0.003 |

Notes: HR: hazard ratio; CI: confidence interval.

Supplemental Table 2. Multivariate survival analysis of the patients

|  | **HR** | **Lower** | **Upper** | ***P* Value** |
| --- | --- | --- | --- | --- |
| ***Step 1*** |  |  |  |  |
| Gender | 1.675 | 0.252 | 11.262 | 0.593 |
| Age | 0.968 | 0.885 | 1.058 | 0.471 |
| Serum creatinine | 0.996 | 0.991 | 1.002 | 0.162 |
| Anticardilolipin antibody | 0.456 | 0.068 | 3.055 | 0.419 |
| SLEDAI | 0.928 | 0.81 | 1.062 | 0.277 |
| Anemia | 0.151 | 0.024 | 0.955 | 0.045 |
| Plasmapheresis | 33.239 | 2.144 | 515.392 | 0.012 |
| ***Step 2*** |  |  |  |  |
| Gender | 0.64 | 0.17 | 2.407 | 0.509 |
| Age | 0.96 | 0.9 | 1.024 | 0.214 |
| Serum creatinine | 0.998 | 0.994 | 1.002 | 0.253 |
| SLEDAI | 1.002 | 0.918 | 1.095 | 0.956 |
| Anemia | 0.262 | 0.075 | 0.915 | 0.036 |
| Plasmapheresis | 9.114 | 1.838 | 45.199 | 0.007 |
| ***Step 3*** |  |  |  |  |
| Gender | 0.568 | 0.19 | 1.701 | 0.312 |
| Age | 0.958 | 0.901 | 1.019 | 0.172 |
| Serum creatinine | 0.999 | 0.995 | 1.002 | 0.368 |
| Anemia | 0.196 | 0.063 | 0.608 | 0.005 |
| Plasmapheresis | 10.16 | 2.633 | 39.202 | 0.001 |
| ***Step 4*** |  |  |  |  |
| Gender | 0.545 | 0.188 | 1.578 | 0.263 |
| Age | 0.956 | 0.901 | 1.015 | 0.14 |
| Anemia | 0.186 | 0.062 | 0.559 | 0.003 |
| Plasmapheresis | 7.792 | 2.42 | 25.087 | 0.001 |
| ***Step 5*** |  |  |  |  |
| Anemia | 0.244 | 0.091 | 0.649 | 0.005 |
| Plasmapheresis | 8.914 | 3.028 | 26.247 | <0.001 |

Notes: HR: hazard ratio; CI: confidence interval.
